# Management of BRCA Tumour Testing in an Integrated Molecular Tumour Board Multidisciplinary Model

**DOI:** 10.3389/fonc.2022.857515

**Published:** 2022-04-08

**Authors:** Jacopo Azzollini, Andrea Vingiani, Luca Agnelli, Elena Tamborini, Federica Perrone, Elena Conca, Iolanda Capone, Adele Busico, Bernard Peissel, Erica Rosina, Monika Ducceschi, Mara Mantiero, Salvatore Lopez, Francesco Raspagliesi, Monica Niger, Matteo Duca, Silvia Damian, Claudia Proto, Filippo de Braud, Giancarlo Pruneri, Siranoush Manoukian

**Affiliations:** ^1^ Unit of Medical Genetics, Department of Medical Oncology and Hematology, Fondazione IRCCS Istituto Nazionale dei Tumori, Milan, Italy; ^2^ Department of Pathology and Laboratory Medicine, Fondazione IRCCS Istituto Nazionale dei Tumori, Milan, Italy; ^3^ Oncology and Hemato-oncology Department, University of Milan, Milan, Italy; ^4^ Medical Oncology Department, Fondazione IRCCS Istituto Nazionale dei Tumori, Milan, Italy; ^5^ Department of Gynecologic Oncology, Fondazione IRCCS Istituto Nazionale dei Tumori, Milan, Italy

**Keywords:** ovarian cancer, homologous recombination, HBOC (hereditary breast and ovarian cancer), genetic counselling, BRCA1, BRCA2, somatic variants

## Abstract

Tumour testing of the *BRCA1/2* genes is routinely performed in patients with different cancer histological subtypes. To accurately identify patients with tumour-detected germline pathogenic variants (PVs) is a relevant issue currently under investigation. This study aims at evaluating the performance of the tumour-to-germline diagnostic flowchart model defined at our Institutional Molecular Tumour Board (MTB). Results from tumour BRCA sequencing of 641 consecutive unselected cancer patients were discussed during weekly MTB meetings with the early involvement of clinical geneticists for appropriate referral to genetic counselling. The overall tumour detection rate of *BRCA1/2* PVs was 8.7% (56/641), ranging from 24.4% (31/127) in high-grade ovarian cancer to 3.9% (12/304) in tumours not associated with germline *BRCA1/2* PVs. Thirty-seven patients with PVs (66%) were evaluated by a clinical geneticist, and in 24 of them (64.9%), germline testing confirmed the presence of the PV in blood. Nine of these patients (37.5%) were not eligible for germline testing according to the criteria in use at our institution. Cascade testing was subsequently performed on 18 relatives. The tumour-to-germline diagnostic pipeline, developed in the framework of our institutional MTB, compared with guideline-based germline testing following genetic counselling, proved to be effective in identifying a higher number of germline BRCA PVs carriers.

## Introduction

Genomic analysis of tumour DNA is mandatory for both the pathological diagnosis and the selection of patients who may benefit from drugs targeting specific gene aberrations (1, 2). In the last years, next-generation sequencing (NGS) techniques for tumour testing is increasingly used as a clinical tool to simultaneously analyze hundreds of genes in different cancer settings (3–5). This approach fostered the acquisition of a massive amount of tumour molecular data and increased the detection of variants in cancer-predisposing genes. These identified in tumours, although not necessarily actionable in terms of therapeutic targeting, might be of germline origin and thus particularly relevant to address tumour risks in patients and their relatives (6–8).

Among high-penetrance cancer-predisposing genes, pathogenic variants of *BRCA1* and *BRCA2* are the most frequent germline alterations found at tumour testing and represent the most common incidental germline finding in unselected populations of cancer-affected individuals (9–11). Different approaches have been proposed to identify carriers of germline variants among individuals undergoing tumour testing, including parallel tumour and germline analysis (parallel testing) or upfront tumour sequencing followed by germline testing in selected cases (tumour-to-germline testing) (12–14). Data on the diagnostic pipeline, particularly in the Italian population, are currently scarce and mostly limited to OC patients (13, 15–17).

Herein we present the results of a tumour-to-germline model of BRCA testing developed by an Institutional Molecular Tumour Board (MTB) in a large Italian cohort of consecutive patients with different cancer histotypes, providing data on its impact on genetic counselling and cascade testing in family members.

## Materials and Methods

### Patients Cohort

The study cohort was selected among consecutive cancer-affected individuals who underwent genetic testing on tumour DNA for therapeutic purposes, regardless of the type of cancer, age at diagnosis and family history. We included in the analysis only patients discussed at the Molecular Tumour Board (MTB) of the Fondazione IRCCS Istituto Nazionale dei tumori of Milan (INT) between April 29^th^ 2020 and June 30^th^ 2021, and for whom successful complete sequencing of the *BRCA1* and *BRCA2* genes in the tumour was available. Patients in the final cohort were either affected with tumours associated with BRCA defects, which might be eligible for treatment with PARP inhibitors (PARPi), or developed tumours with indications for treatments targeting different molecular pathways. Genetic testing in the first group was carried out mainly with NGS of only the BRCA genes, while in the second group the analysis was performed through a larger gene panel.

### Tissue Preparation and Tumour Sequencing

In 185 out of 641 patients, mostly affected with ovarian cancer, tumour variants of the *BRCA1* and *BRCA2* genes were assessed by in-house NGS testing using the Oncomine BRCA Research Assay (Thermo Fisher Scientific, Inc). This assay provided a 100% coverage of all *BRCA1* and *BRCA2* exons, with an average of 64 bases of intronic flanking sequences upstream and downstream of each exon. Five µm sections from formalin-fixed paraffin-embedded (FFPE) samples were manually microdissected to isolate the highest percentage of neoplastic cells. Genomic DNA was extracted with protease K (incubation ON at 55°C) and quantified with Qubit dsDNA BR kit (Thermo Fisher Scientific, Inc).

The libraries were prepared with the IonAmpliSeq Library kit 2.0 (Thermo Fisher Scientific, Inc) and quantified with Qubit dsDNA HS kit (Thermo Fisher Scientific, Inc) following the manufacturer’s instructions. The libraries are diluted to 25 pm, pooled and loaded on the Ion Chef to perform emulsion PCR and chip loading, using Ion 318 v2 Chip and ION PGM HI-Q view Chef kit, according to the manufacturer’s instructions.

Data were processed using the Torrent Suite. Variant calling from sequencing data was generated by the Variant Caller plugin. To eliminate erroneous base calling, we set each variant coverage >40, a variant frequency on each sample >5 and a quality value >30. The resulting variants were annotated using the Ensemble Variant Effect Predictor pipeline, Ion Reporter analysis software. Each variant is displayed on IGV2.11.1. Among the called variants, synonymous variants are filtered out and other variants are classified into pathogenicity classes according to the Evidence-based Network for the Interpretation of Mutant Alleles (ENIGMA) consortium guidelines (https://enigmaconsortium.org/). This assay could not reliably detect large intragenic rearrangements.

The other 456 patients were mainly affected with tumours for which PARPi are not indicated, but other therapeutic options targeting different genes and molecular pathways are available. This group underwent quantitative NGS on tumour FFPE slide specimens using the validated Foundation One CDx assay (Foundation Medicine, Cambridge, MA), which detects nucleotide substitutions, small insertions/deletions and copy number alterations in 324 genes (18). This assay provided also an estimate of the tumour mutational burden (TMB) in 364 samples.

### Molecular Tumour Board Workflow

**Figure 1:**
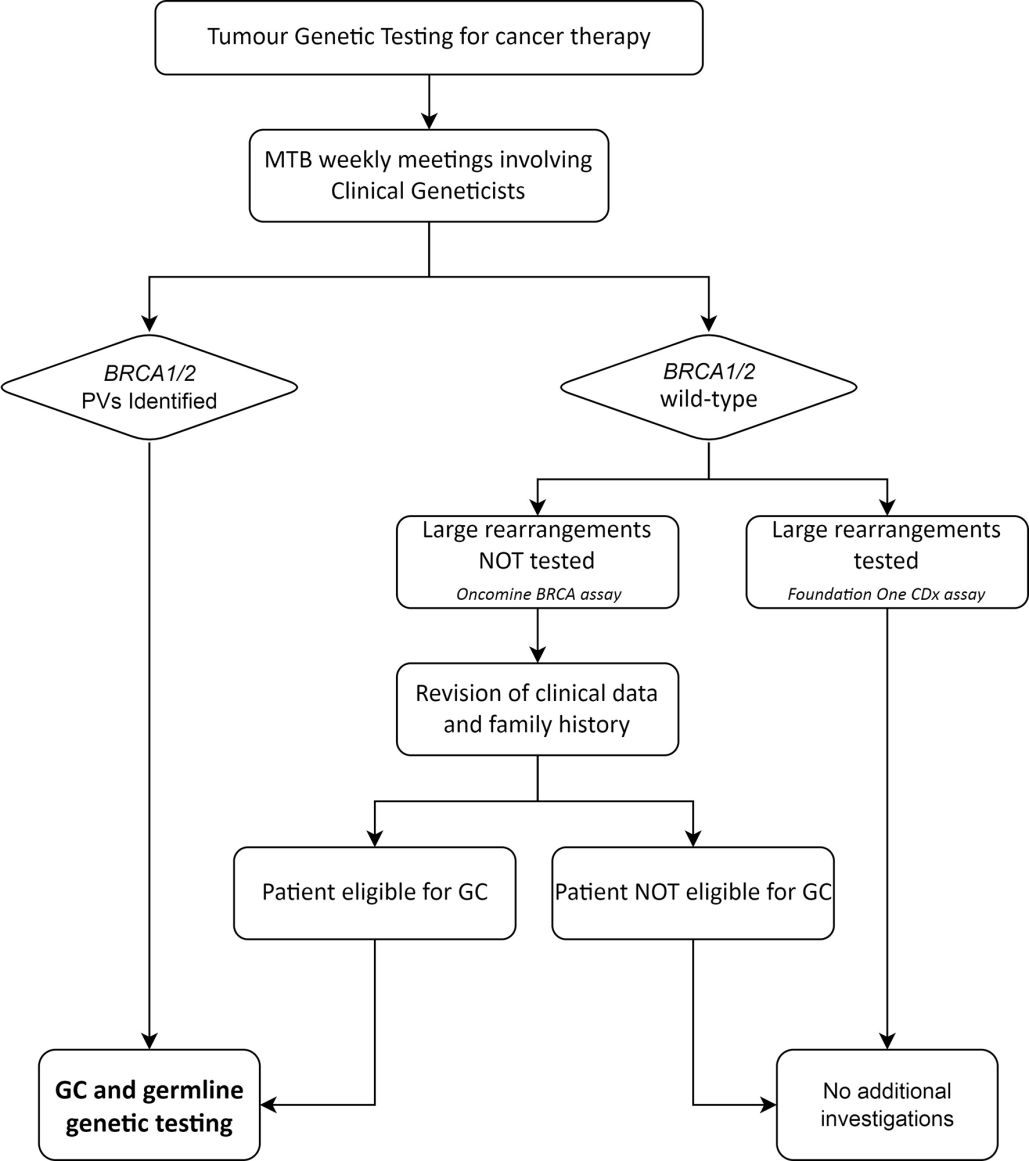
Workflow of the management of BRCA tumour testing results with potential germline implications. MTB, Molecular Tumour Board; GC, genetic counselling; PVs, pathogenic/likely pathogenic variants.

The MTB, composed of pathologists, oncologists, molecular biologists, bioinformatics and clinical geneticists, performed a weekly evaluation of the molecular analyses, interpreted and classified the identified genetic variants and provided recommendations on therapeutic targets and the appropriateness of genetic counselling and additional genetic testing. In case pathogenic or likely pathogenic *BRCA1/BRCA2* variants (henceforth termed PVs), as defined according to the ENIGMA criteria, were identified, clinicians were advised by the clinical geneticists of the MTB to refer the patients to genetic counselling for targeted germline testing. Information about clinical or family features suggestive of Hereditary Breast and Ovarian Cancer syndrome (HBOC) were reviewed by the clinical geneticists in all patients affected with a BRCA-associated tumour. Since the Oncomine BRCA assay does not detect large rearrangements, all patients, who tested negative at this assay yet fulfilled the empirical selection criteria for BRCA germline testing used at our Institution (**Supplementary Table 1**) (19), were referred to genetic counselling and were offered the germline analysis of large rearrangements involving the *BRCA1*/*2* genes (**Figure 1**).

If a PV was confirmed to be germline, the probands were informed about the opportunity of genetic counselling and cascade testing in family members.

In case PVs affecting actionable genes other than *BRCA1* and *BRCA2* were identified at the Foundation One CDx assay, genetic counselling and germline testing were offered according to the ESMO Precision Medicine Working Group recommendations (20).

The Local Ethics committee approved the use of both clinical and molecular data collected by the MTB for clinical studies and granted exemption from requiring written consent for tumour genetic testing from the patients, as these analyses were conducted in a routine diagnostic and care setting (approval number INT 227/20). All the probands and relatives who underwent germline testing were aged over 18 and provided signed informed consent for the use of their biological samples and data for both diagnostic and research purposes. The consent form was in use at our Institution and was previously approved by the INT Ethics committee (INT 171/15).

### Germline Testing

Two EDTA tubes of peripheral blood samples were collected from each patient who performed genetic counselling and was eligible for germline testing. Whole blood DNA was isolated through the MagCore^®^ Super automatic workstation with the MagCore^®^ Genomic DNA Whole Blood Kit (Diatech LabLine SRL, Jesi, Italy).

Targeted Sanger sequencing of tumour-detected *BRCA1/BRCA2* PVs was performed on purified PCR products by using BigDye^®^ Terminator v.3.1 Cycle Sequencing kit (Thermo Fisher Scientific, Inc.) and run on 3730Xl DNA Analyzer (Applied Biosystems; Thermo Fisher Scientific, Inc.), after purification with Agencourt CleanSeq^®^-Beckman Coulter. Sequences were analyzed by Mutation Surveyor^®^ Software (v5.0.1; SoftGenetics, LLC., State College, PA, USA). Targeted sequencing results were confirmed on both blood aliquots collected from each patient. Variants of uncertain clinical significance identified at tumour testing were not systematically investigated at the germline level.

Eligible probands, who resulted negative at tumour testing with the Oncomine BRCA assay, were analysed for large deletions and duplications of *BRCA1* and *BRCA2* on blood DNA with the SALSA MLPA kits P045 *BRCA2*/*CHEK2* and P002 *BRCA1* probe mix (MRC-Holland, Amsterdam, the Netherlands), following the manufacturer’s instructions. MLPA products were run on the 3730Xl DNA Analyzer (Applied Biosystems; Thermo Fisher Scientific, Inc.) with the Gene Mapper Module (Applied Biosystems; Thermo Fisher Scientific, Inc.). The results were analyzed through the Gene Marker Software v2.7.0 (SoftGenetics, LLC, State College, PA, USA).

### Statistical Analysis

Descriptive statistics and confidence intervals of the mean were used for reporting the age at tumour testing of the patients. The two-tailed Fisher’s exact test was employed to compare the frequency of BRCA PVs in different tumour types. An unpaired *t-*test was used to compare the age at diagnosis of OC between patients with germline versus somatic only BRCA PVs. One-sided Mann Whitney test for unpaired data was used to compare the tumour mutational burden (TMB) values between BRCA wild-type tumours and tumours with BRCA PVs and to compare the allele frequency of germline versus somatic only PVs.

## Results

### Patients

The study population was represented by all the patients consecutively analyzed by NGS and discussed by the Institutional Molecular Tumour Board between April the 29^th^, 2020 and June the 30^th^, 2021. Overall, 1549 patients were evaluated, including 840 females and 709 males, with a median age at tumour testing of 64 years (range 0 – 90 years). Tumour sequencing of all coding exons of the *BRCA1* and *BRCA2* genes was carried out in 641 (41.3%) cases, including patients with ovarian (OC, 154, 24%), biliopancreatic (BPC, 135, 21.1%), breast (BC, 36, 5.6%) and prostate (PC, 12, 1.9%) cancer, as well as other tumours not typically associated with BRCA1/2 PVs, including gastrointestinal (GI) tumours (147, 22.9%) and other histotypes (157, 24.5%). The histology of OC patient tumours was high-grade serous (HGSOC, 112 cases), high-grade non-serous (HGOC, 15 cases) and low grade or unknown/uncertain type (27 cases) carcinoma. Out of the 641 patients tested for BRCA, 185 (128 of which were OC) were analysed using the Oncomine BRCA Research Assay, and the remaining 456 (26 OCs, 25 BCs, 106 BPCs, 5 PCs, 146 GI tumours, and 148 other tumours), using the Foundation One CDx assay.

### BRCA Tumour Testing Results

Out of 641 patients analyzed, 56 (8.7%, 20 in *BRCA1* and 36 in *BRCA2*) bore tumour BRCA PVs and 64 variants of uncertain significance (VUS). All the PVs were small truncating variants, except two large deletions at BRCA2, representing large intragenic rearrangement, which were both detected using the Foundation One assay. A complete list of the detected PVs is available in **Supplementary Table 2**.

**Figure 2:**
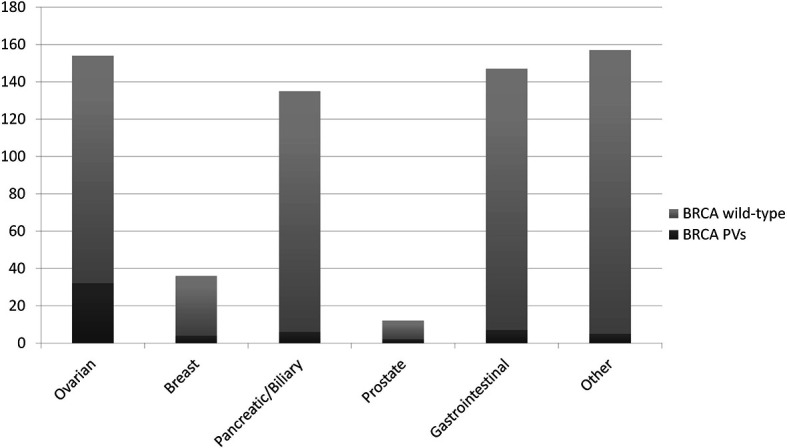
Detection rate of BRCA pathogenic/likely pathogenic variants (PVs) at tumour testing in different primary tumours.

The detection rate of PVs (DR) in the whole cohort was 8.7% (56/641). A wide range of DRs was found across the histotypes analysed: 20.8% in OC patients (32/154, 16 *BRCA1* and 16 *BRCA2*, rising to 24.4% in HGOCs), 11.1% in BC patients (4/36, 2 *BRCA1* and 2 *BRCA2*), 4.4% in BPC patients (6/135, *BRCA2*), 16.7% in PC patients (2/12, *BRCA2*), 4.8% in GI tumour patients (7/147, 2 *BRCA1* and 5 *BRCA2*), and 3.2% in patients with other tumours (5/157, *BRCA2*) (**Figure 2**).

As expected, the DR in OCs was significantly higher compared with all other tumour types considered together (*p<0.0001*). Due to the limited number of BCs and prostate cancers in our cohort, we could not detect a significant difference in the DR in these compared with other tumours. Notably, in BPC the DR was not different compared with tumours not in the spectrum of HBOC.

**Figure 3:**
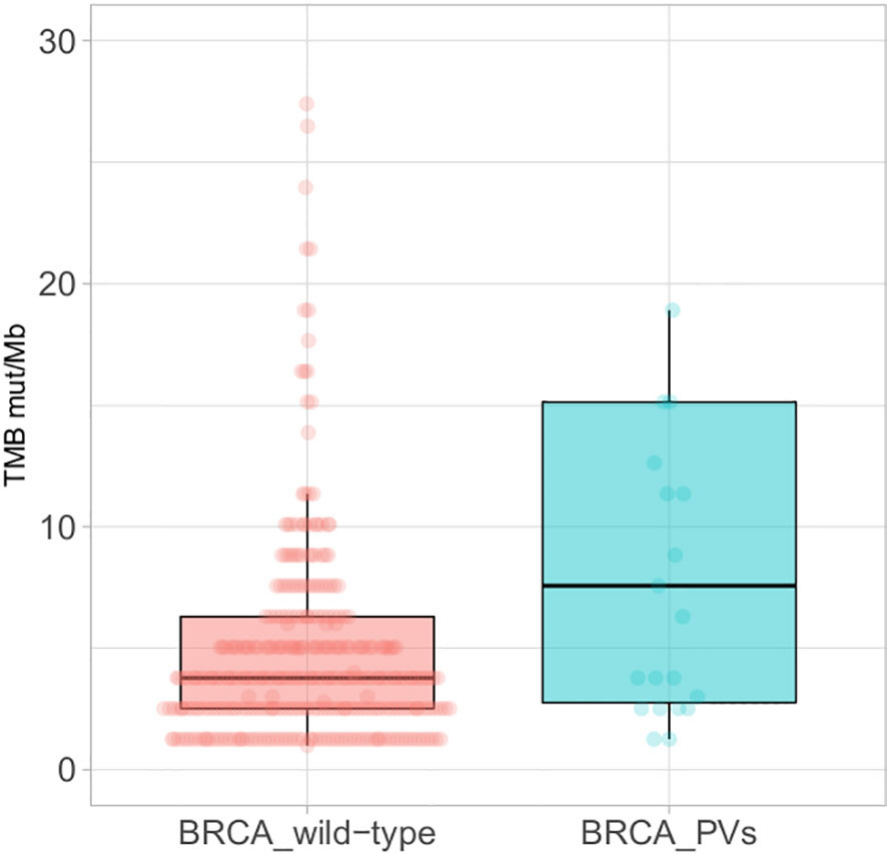
Boxplots of the Tumour Mutational Burden (TMB) in BRCA wild-type tumours (left) and tumours with BRCA pathogenic/likely pathogenic variants (PVs) (right); extreme outliers (TMB >30 mut/Mb) are not displayed.

The tumour mutational burden (TMB) was available in 364 of the 456 cases analysed, including 23 tumours with BRCA PVs, and it was significantly higher in BRCA mutated (median 7.57 mut/Mb) than wild-type tumours (median 3.78 mut/Mb) (*p<0.005*) (**Figure 3**).

### Referral to Genetic Counselling and Germline Testing Results

Seven out of the 56 patients with a PV in the tumour had previously undergone germline testing following a clinical geneticist evaluation, and five of them yielded consistent results. In one of the two OC patients with discordant results, the c.1813delA PV, located at a homopolymeric region of *BRCA2*, was not detected by the Oncomine variant calling software. Nevertheless, a subsequent manual curation analysis with IGV tools allowed us to demonstrate that the variant was occurring in the tumour as well. In the other patient, bearing the germline *BRCA2* c.4284dupT p.(Gln1429Serfs*9) variant, tumour testing on relapsing OC tissue showed the large *BRCA2* deletion p.(Leu1334_Asn1742del) encompassing the germline variant. This patient showed a clinical progression upon platinum and PARPi treatment, suggesting the occurrence of a resistance reverse mutation at the tumour level.

**Figure 4:**
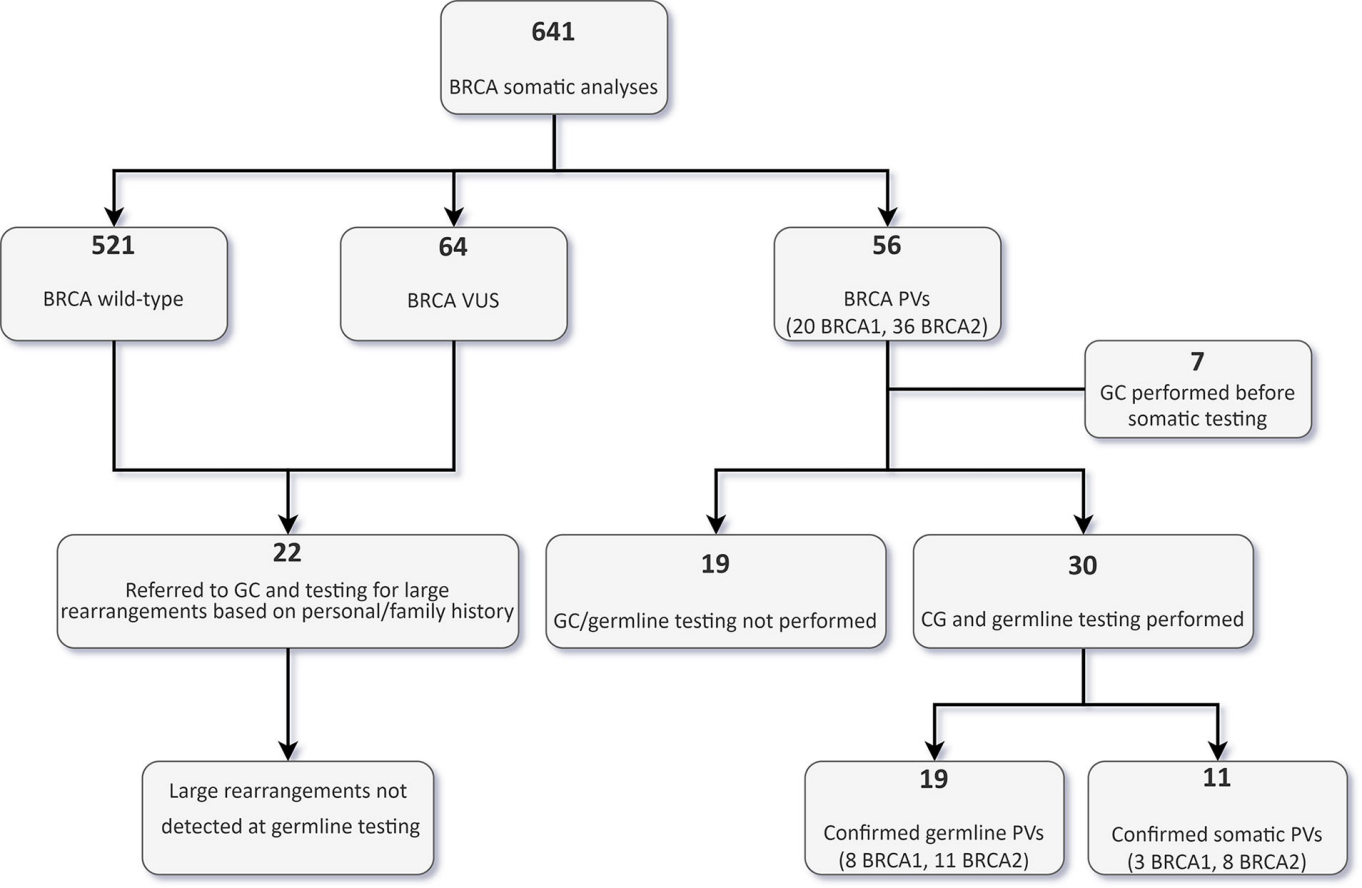
Flowchart of tumour and germline BRCA testing results in the whole cohort. VUS, variants of uncertain significance; GC, genetic counselling; PVs, pathogenic/likely pathogenic variants.

Thirty out of the 56 patients with PVs detected by tumour testing were subsequently evaluated by a clinical geneticist and underwent targeted germline testing (**Figure 4**).

**Figure 5:**
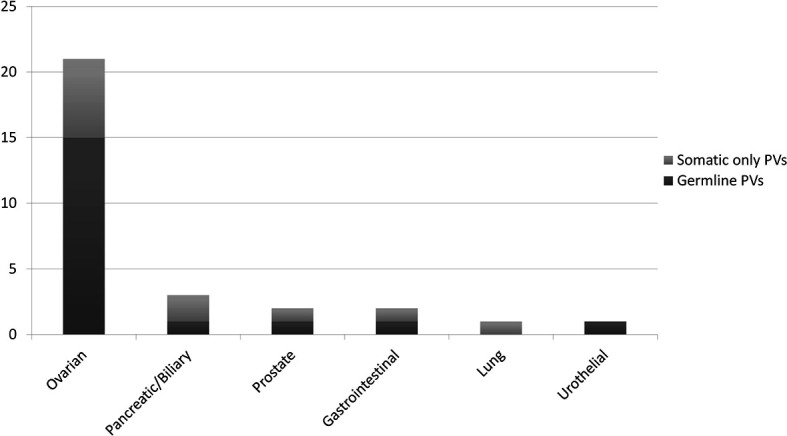
Ratio of somatic only vs. germline BRCA pathogenic/likely pathogenic variants (PVs), according to the primary tumour type, in 30 patients who underwent both tumour and germline testing.

In 19 patients (15 OC, and one each BPC, duodenal cancer, PC and urothelial cancer patients), the PV was confirmed to be germline. Nine of these 19 patients (47.4%) would not have been eligible for germline testing based on the clinical criteria in use at our Institution. With regards to OC patients, 33.3% of germline carriers (5/15) would not have been tested if the tumour sequencing had not been performed frontline. Conversely, of the 30 patients who underwent germline analysis, 11 (36.7%) had somatic only PVs, and nine (81.9%) of them would not have been eligible for germline testing. In the OC patient subgroup with a BRCA PV in the tumour, 28.6% (6/21) of the PVs were not detected at germline testing (**Figure 5**).

Age at diagnosis of OC in patients with germline PVs was lower than in patients with somatic only PVs (53 vs 57.5 years), although the difference did not achieve statistical significance (**Supplementary Figure 1**). Median PV allele frequency, available in 23 cases, was 0.69 (range 0.39-0.94) for germline variants and 0.19 (range 0.07-0.61) for somatic variants (*p<0,001*). Although mutational tumour burden data were available for eight patients with germline PVs only, no difference was observed between BRCA-mutated and wild-type tumours (median 3.78 mut/Mb, *p=n.s.*).

Nineteen of the 56 patients bearing tumour BRCA PVs did not perform genetic counselling at our Institution due to different reasons, including loss at follow-up, death or severe health condition, or refusal to perform germline testing. No further BRCA aberrations were detected by Multiplex Ligation-dependent Probe Amplification (MLPA) in 22 patients (18 OC and 4 BC patients) eligible for germline testing due to personal or family history but testing negative at the Oncomine BRCA assay (**Figure 4**).

### Genetic Counselling and Germline Testing in Family Members

**Figure 6:**
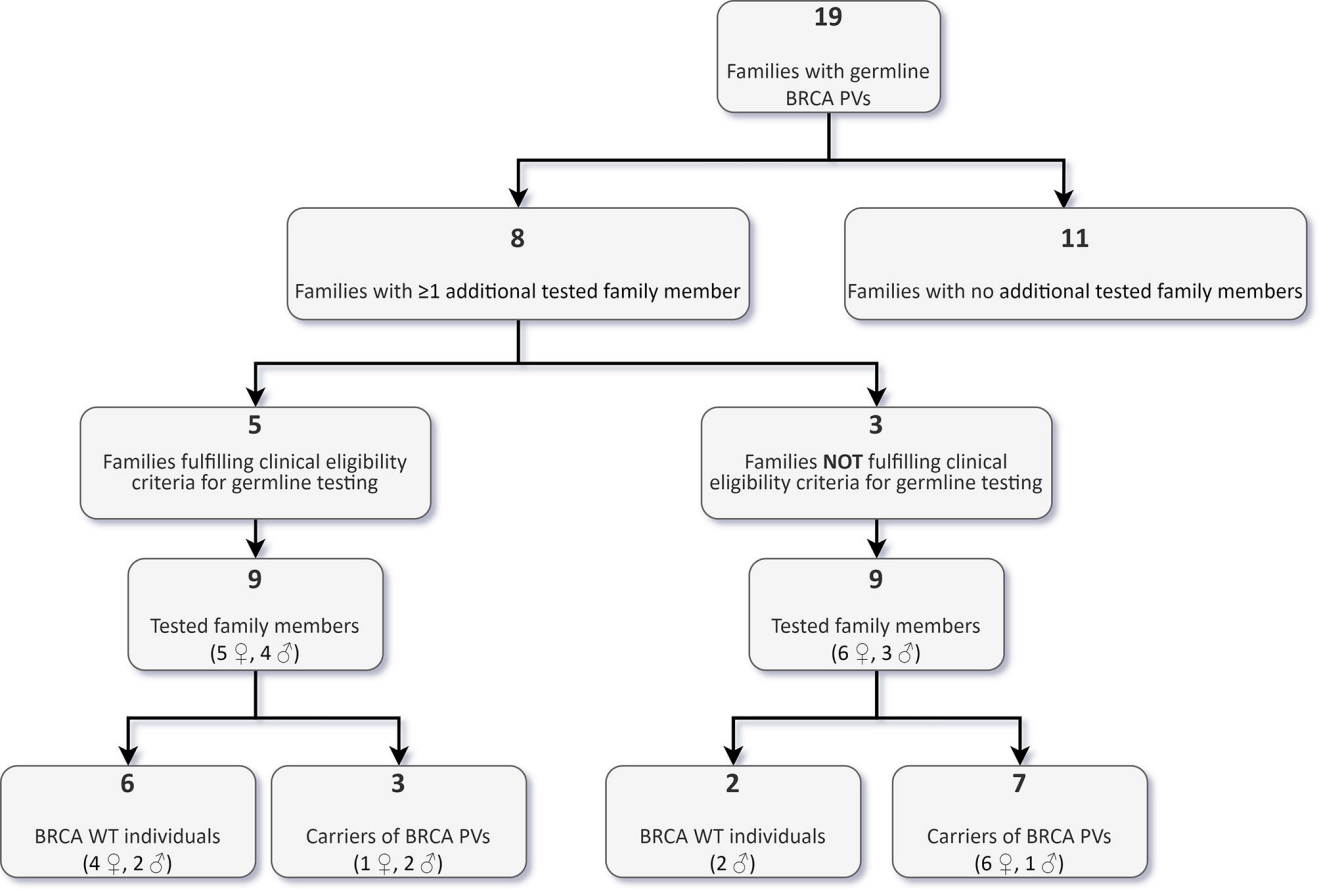
Targeted analysis of BRCA pathogenic/likely pathogenic variants (PVs) in family members of carriers identified through tumour testing.

Following the identification of a BRCA germline PV in 19 probands, we offered genetic counselling and cascade testing to family members. As of the 31^st^ October 2021, 18 individuals from eight families (11 females, 7 males), with a median age of 55 years (range 33-77 years), performed targeted germline testing, and ten were found to have inherited the family variant (**Figure 6**).

Nine of these carriers reported a negative personal oncological history at the time of counselling, while one had developed colon cancer at 64 years. Among the eight non-carriers, two reported a personal history of cancer, one with bilateral BC at the age of 52 (right side) and 53 years (left side), the other with a testicular embryonal carcinoma at the age of 26 years. Nine (50%) of the 18 tested family members, including seven carriers, belonged to three families, which would have not been eligible for germline testing according to institutional clinical criteria.

## Discussion

Therapies targeting homologous recombination defects (HRD) are steadily adopted in different cancer settings, yielding a higher volume of BRCA tumour testing carried out in pathology labs. Recently, the updated *post-hoc* analysis of progression-free survival from SOLO1 trial in germline and somatic BRCA mutated advanced OC patients reported after 5 years of follow-up a median progression-free survival of 56.0 months with olaparib versus 13.8 months with placebo (21).

Along this line, several trials are ongoing for addressing whether, in addition to germline PVs, somatic BRCA mutations may also be predictive of PARPi efficacy in several cancer settings. The identification of BRCA as an actionable target for personalized therapy revolutionized its testing workflow, which was traditionally preceded by genetic counselling, and significantly increased the volume of patients analyzed (22, 23)

In this complex scenario, BRCA testing is recommended to be managed by a multidisciplinary team of professionals, including clinical geneticists, that takes care of variant annotation, clinical trial recruitment and potential association with germline variants. In this study, we reported from a clinical geneticist perspective the BRCA diagnostic flowchart implemented by our Institutional MTB in an Italian cohort of 641 cancer patients genomically profiled by targeted NGS.

In the time frame of the study, 641 tumours underwent full sequencing of the BRCA genes. About one-fifth of these were OCs, which were mostly analysed with the Oncomine BRCA assay (83%, 128/154). Conversely, most other tumours underwent sequencing with the larger Foundation One multigene panel (88%, 430/487). The latter group included also tumours for which PARPi are not indicated, but other therapeutic options targeting different molecular defects are available.

In accordance with previous data, the frequency of tumour BRCA PVs was roughly 20% in OC (13, 24–27) and 4% in BPC (28). Notably, in our cohort, the DR in BPC was not different compared with tumours not in the spectrum of HBOC, possibly due to the relatively small number of patients investigated. However, it has been proposed that outside OC and BC settings, a higher fraction of BRCA PVs might represent an incidental finding, without a causal connection to tumour pathogenesis. This hypothesis is supported by the study by Yost et al, who reported a low frequency of both PVs and loss-of-heterozygosity (LOH) events involving the BRCA genes in tumours other than OC and BC (26).

In our model, targeted germline testing is offered whenever a BRCA PV is identified in the tumour, irrespective of the histological subtype, allele frequency and TMB. Although an allele frequency close to or higher than 50% could point to a germline origin of the variant (12, 20), as confirmed in our series, multiple factors may affect its estimate, including tumour cellularity, copy-number variation (CNV) and LOH, as well as sequencing and variant calling methods used for the tumour analysis (10, 29). A high TMB is another feature that could be associated with BRCA PVs. As previously reported for several cancer types (28, 30, 31), BRCA PVs in our cohort were associated with a higher TMB. However, our data on the TMB in germline carriers are limited and do not allow us to explore putative differences between germline and somatic only PVs.

A relevant aspect of all the diagnostic flowcharts is represented by the actual concordance between tumour and germline testing. Previous studies evidenced discrepancies between these analyses, mainly due to the low sensitivity in detecting large rearrangements from highly fragmented DNA obtained from FFPE tissues. In a cohort of high-grade epithelial OC patients, Chandrasekaran and colleagues recently reported a 1,4% discordant rate in detecting large rearrangements, due to tumour testing failure (13), thus suggesting performing concurrent/parallel tumour and germline analysis would be preferable. However, other studies showed that the prevalence of large BRCA rearrangements in other tumour types seems to be lower than that observed in OCs. In a large cohort from the US, Sharder et al. identified 52 carriers of BRCA PVs and only two large BRCA deletions among 1566 patients with advanced neoplasms (9). This finding was subsequently confirmed by Mendelker and colleagues, who reported 59 carriers of BRCA PVs and only one individual with a deletion, affecting exon 8 of *BRCA1*, among 1040 germline analyses of patients with unselected advanced cancers (7). In addition, the parallel tumour and germline approach, besides being less affordable in terms of costs, has also the disadvantage of waiving the choice of whether or not to undergo predictive germline testing. In our cohort, almost one-third of patients in whom a BRCA PV had been identified in the tumour, despite being referred to genetic counselling, were lost to follow up. A subset of these did not desire to know their germline status. Another potential limit could be to generate additional anxiety in patients whose main focus is the treatment of the present tumour rather than future risks for themselves or their relatives.

Other authors proposed a tumour-to-germline approach based on tumour sequencing up front, followed by targeted germline testing in selected patients. A recent study provided evidence that discussing molecular results of tumour testing within a MTB including clinical geneticists improved both the referral to genetic counselling and the identification of germline PVs (12). In our tumour-to-germline flowchart, all the molecular results along with clinical and familial data of patients with negative tumour testing are reviewed by clinical geneticists. All the patients eligible for germline testing are offered an MLPA analysis when the techniques used for tumour sequencing were inadequate for detecting large rearrangements. In this regard, out of the 22 patients with wild-type tumour BRCA analyzed by MLPA, no one showed gene rearrangement. Overall, only one large rearrangement was detected in 181 patients with tumours in the spectrum HBOC (including BC, OC, BPC and PC), suggesting that the probability of overlooking these molecular defects is seemingly low also in our series.

However, there are limitations in the use of tumour testing for guiding germline analyses. In our series, one patient previously found to be a carrier of a germline *BRCA2* PV was negative at tumour testing, likely since it was located in a homopolymeric region, which is usually inefficiently sequenced by Ion torrent-based techniques. However, the frequency and impact of this phenomenon have not yet been determined (32–34).

An additional potential flaw of tumour testing is represented by the detection of reverse mutation. In an OC patient with acquired resistance to both platinum salts and PARPi, we found an in-frame deletion of 409 codons affecting BRCA2 exon 11 in tissue obtained from relapse. A former analysis had revealed a germline pathogenic *BRCA2* point variant within the same region of the somatically deleted allele, allowing to classify the deletion as a reverse mutation. In this case, if a complete germline analysis had not been performed, the tumour testing result would have been misinterpreted, with a potentially negative impact on the patient’s treatment. Furthermore, the germline variant would not have been recognized, with consequences on both the patient and her relatives. Although this phenomenon has been consistently reported in BRCA-related tumours (35, 36), its occurrence is uncommon in untreated primary cancers, suggesting that testing for targetable BRCA PVs should be carried out in the most recent specimen available. Finally, it has to be underlined that reverse mutations might mask underlying germline PVs when tumour testing is carried out in tumours previously exposed to cytotoxic agents, which might have selected clones with reverse mutations conferring drug resistance.

Collectively, these data suggest that the risk of missing BRCA PVs with a tumour-to-germline approach would be limited. In addition, also parallel tumour and germline testing would still overlook a fraction of either somatic or germline pathogenic alterations, such as epigenetic events (37–39).

Using our tumour-to-germline diagnostic flowchart, we identified an additional 50% of germline carriers. In particular, 9 out of 19 germline carriers of BRCA PVs would not have been eligible for germline testing according to the institutional criteria based on personal or family history. Interestingly, the frequency of these incremental germline PVs in our cohort is in line with that reported by Samadder and colleagues in a recent study, which compared universal germline testing of cancer affected individuals versus guideline-directed targeted testing (11). On this basis, it could be argued that universal germline testing could be proposed as an alternative screening method to the herein presented tumour-to-germline diagnostic flowchart. However, albeit it is seemingly as effective in identifying germline carriers, this approach would overlook a considerable fraction of patients with PVs confined to the tumour, who might still benefit from cancer-specific targeted therapies (e.g. PARPi in OC).

Although our model has the overt advantage of identifying a higher number of individuals at increased genetic risk, it has also generated some concerns in our clinical practice. By the end of October 2021, an equal number of relatives from families eligible and non-eligible for germline testing, based on the empirical criteria in use at our Institution, underwent genetic counselling and targeted testing. However, in some patients with anamnestic features not suggestive of a genetic predisposition, we experienced more pronounced distress and lower acceptance of a positive test result, which might, in turn, lead to lower compliance with the recommended preventive measures. Moreover, indications on both screening and risk-reducing surgery are currently provided considering risk estimates calculated on high-risk families (i.e. with multiple affected individuals). Recent population-based case-control studies demonstrated that the penetrance of BRCA PVs is lower in unselected cohorts (40, 41), likely due to the influence of polygenic inheritance in certain families, with multiple susceptibility loci that modify the penetrance of BRCA PVs (42). Consequently, there is a potential risk of “overtreatment”, due to an overestimation of the risks in patients and relatives from non-eligible families. Obtaining more accurate and tailored penetrance estimates based on larger cohorts and population-specific studies will be critical, along with the future inclusion of personal polygenic risk scores in a diagnostic setting.

To the best of our knowledge, this is the first study to describe a model in which all the tumour testing results are reviewed by the MTB with the early involvement of clinical geneticists. The presence of oncogenetics experts in the early phases enhanced the annotation and interpretation of suspected germline variants. Moreover, compared with the “mainstreamed genetic testing” strategy, it allowed a more direct and prompt referral to genetic counselling for both patients with PVs and individuals who should undergo further germline investigations irrespective of the tumour testing results. Since this approach proved to be feasible and effective in the management of HBOC patients, we expect it to be readily applied also to other cancer-predisposing syndromes.

## Conclusion

The herein discussed model and diagnostic flowchart of our Institutional MTB proved to be effective in identifying a higher number of germline carriers of BRCA PVs compared with guideline-based germline testing following genetic counselling. Since most targeted therapies are approved only for patients with germline PVs, assessing the germline status is mandatory for guiding treatment. However, our data highlight that, compared with parallel tumour and germline testing, an approach based on tumour sequencing upfront with the early involvement of clinical geneticists is feasible to enhance the identification of germline carriers without increasing the burden on genetic counselling services.

Overall, we also observed good compliance of both patients and clinicians involved in their medical management. Moreover, this model promoted interdisciplinary discussion, which is of paramount importance to enhance precision healthcare for patients and their families. The collection of data on a larger cohort of patients will provide further insight into our observations and better address points of improvement of the presented model.

## Data Availability Statement

The raw data supporting the conclusions of this article will be made available by the authors, without undue reservation.

## Ethics Statement

The studies involving human participants were reviewed and approved by Ethics committee of the Fondazione IRCCS Istituto Nazionale dei Tumori, Milan, Italy. Written informed consent for participation was not provided by the participants’ legal guardians/next of kin because: The Ethics committee of the Fondazione IRCCS Istituto Nazionale dei Tumori di Milano (INT) approved the use of both clinical and molecular data collected by the MTB for clinical studies and granted exemption from requiring written consent for tumour genetic testing from the patients, as these analyses were conducted in a routine diagnostic and care setting (approval number INT 227/20). All the probands and relatives who underwent germline testing were aged over 18 and provided signed informed consent for the use of their biological samples and data for both diagnostic and research purposes. The consent form was in use at our Institution and was previously approved by the INT Ethics committee (INT 171/15).

## Author Contributions

Study concept and design: SM, GP. Acquisition of data: JA, AV, ET, FP, EC, IC, AB, BP, ER, MoD, MM, SL, FR, MN, MaD, SD, CP, FB, GP and SM. Analysis and interpretation of data: JA, ER and SM. Drafting of the manuscript: JA, SM. Critical revision of the manuscript for important intellectual content: All authors. Study supervision: SM, FB and GP. The authors read and approved the final version of the manuscript.

## Funding

Roche Italia SpA, INT protocol 277/20, to GP for the setup of the institutional molecular tumour board platform. The funder was not involved in the study design, collection, analysis, interpretation of data, the writing of this article or the decision to submit it for publication.

## Conflict of Interest

FB: Consultant Advisory Board: Roche, EMD Serono, NMS Nerviano Medical Science, Sanofi, MSD, Novartis, Incyte, BMS, Menarini. Speaker: BMS, Healthcare Research & Pharmacoepidemiology, Merck Group, ACCMED, Nadirex, MSD, Pfizer, Servier, Sanofi, Roche, AMGEN, Incyte, Dephaforum. Principal Investigator for Novartis, F.Hoffmann-LaRoche Ltd, BMS, Ignyta Operating INC, Merck Sharp & Dohme Spa, Kymab, Pfizer, Tesaro, MSD, MedImmune LCC, Exelixis Inc., LOXO Oncology Incorporated, DAICHI SANKIO Dev. Limited, Basilea Pharmaceutica International AG, Janssen-Cilag International NV, Merck KGAA MN: Travel expenses from Celgene, speaker honorarium from Accademia della Medicina and consultant honoraria from EMD Serono, Basilea Pharmaceutica, Incyte and MSD Italia.

The remaining authors declare that the research was conducted in the absence of any commercial or financial relationships that could be construed as a potential conflict of interest.

## Publisher’s Note

All claims expressed in this article are solely those of the authors and do not necessarily represent those of their affiliated organizations, or those of the publisher, the editors and the reviewers. Any product that may be evaluated in this article, or claim that may be made by its manufacturer, is not guaranteed or endorsed by the publisher.

## Supplementary Material

Supplementary Figure 1 Age at diagnosis of ovarian cancer in patients with confirmed germline vs. somatic only BRCA pathogenic/likely pathogenic variants (PVs). The median age is indicated by the horizontal lines; eligibility to germline testing according to the empirical criteria used at our Institution is indicated for each patient.

## References

[1] Marchetti A Barbareschi M Barberis M Buglioni S Buttitta F Fassan M. Real-World Data on NGS Diagnostics: A Survey From the Italian Society of Pathology (SIAPeC) NGS Network. Pathologica (2021) 113:262–71. doi: 10.32074/1591-951X-324 PMC8488986 34463674

[2] Aleksakhina SN Imyanitov EN. Cancer Therapy Guided by Mutation Tests: Current Status and Perspectives. Int J Mol Sci (2021) 22:10931. doi: 10.3390/ijms222010931 34681592 PMC8536080

[3] Horak P Fröhling S Glimm H. Integrating Next-Generation Sequencing Into Clinical Oncology: Strategies, Promises and Pitfalls. ESMO Open (2016) 1:e000094. doi: 10.1136/esmoopen-2016-000094 27933214 PMC5133384

[4] Zehir A Benayed R Shah RH Syed A Middha S Kim HR. Mutational Landscape of Metastatic Cancer Revealed From Prospective Clinical Sequencing of 10,000 Patients. Nat Med (2017) 23:703–13. doi: 10.1038/nm.4333 PMC5461196 28481359

[5] Hyman DM Taylor BS Baselga J. Implementing Genome-Driven Oncology. Cell (2017) 168:584–99. doi: 10.1016/j.cell.2016.12.015 PMC5463457 28187282

[6] Seifert BA O’Daniel JM Amin K Marchuk DS Patel NM Parker JS. Germline Analysis From Tumor-Germline Sequencing Dyads to Identify Clinically Actionable Secondary Findings. Clin Cancer Res (2016) 22:4087–94. doi: 10.1158/1078-0432.CCR-16-0015 PMC4987173 27083775

[7] Mandelker D Zhang L Kemel Y Stadler ZK Joseph V Zehir A. Mutation Detection in Patients With Advanced Cancer by Universal Sequencing of Cancer-Related Genes in Tumor and Normal DNA vs Guideline-Based Germline Testing. JAMA (2017) 318:825–35. doi: 10.1001/jama.2017.11137 PMC5611881 28873162

[8] Huang KL Mashl RJ Wu Y Ritter DI Wang J Oh C. Pathogenic Germline Variants in 10,389 Adult Cancers. Cell (2018) 173:355–70. doi: 10.1016/j.cell.2018.03.039 PMC5949147 29625052

[9] Schrader KA Cheng DT Joseph V Prasad M Walsh M Zehir A. Germline Variants in Targeted Tumor Sequencing Using Matched Normal DNA. JAMA Oncol (2016) 2:104–11. doi: 10.1001/jamaoncol.2015.5208 PMC5477989 26556299

[10] Meric-Bernstam F Brusco L Daniels M Wathoo C Bailey AM Strong L. Incidental Germline Variants in 1000 Advanced Cancers on a Prospective Somatic Genomic Profiling Protocol. Ann Oncol (2016) 27:795–800. doi: 10.1093/annonc/mdw018 26787237 PMC4843184

[11] Samadder NJ Riegert-Johnson D Boardman L Rhodes D Wick M Okuno S. Comparison of Universal Genetic Testing vs Guideline-Directed Targeted Testing for Patients With Hereditary Cancer Syndrome. JAMA Oncol (2021) 7:230–7. doi: 10.1001/jamaoncol.2020.6252 PMC7600058 33126242

[12] Klek S Heald B Milinovich A Ni Y Abraham J Mahdi H. Genetic Counseling and Germline Testing in the Era of Tumor Sequencing: A Cohort Study. JNCI Cancer Spectr (2020) 4:pkaa018. doi: 10.1093/jncics/pkaa018 32596633 PMC7306190

[13] Chandrasekaran D Sobocan M Blyuss O Miller RE Evans O Crusz SM. Implementation of Multigene Germline and Parallel Somatic Genetic Testing in Epithelial Ovarian Cancer: SIGNPOST Study. Cancers (Basel) (2021) 13:4344. doi: 10.3390/cancers13174344 34503154 PMC8431198

[14] Liu YL Stadler ZK. The Future of Parallel Tumor and Germline Genetic Testing: Is There a Role for All Patients With Cancer. J Natl Compr Canc Netw (2021) 19:871–8. doi: 10.6004/jnccn.2021.7044 PMC11123333 34340209

[15] de Jonge MM Ruano D van Eijk R van der Stoep N Nielsen M Wijnen JT. Validation and Implementation of BRCA1/2 Variant Screening in Ovarian Tumor Tissue. J Mol Diagn (2018) 20:600–11. doi: 10.1016/j.jmoldx.2018.05.005 29936257

[16] Rivera D Paudice M Gismondi V Anselmi G Vellone VG Varesco L. Ligurian BRCA Working Group. Implementing NGS-Based BRCA Tumour Tissue Testing in FFPE Ovarian Carcinoma Specimens: Hints From a Real-Life Experience Within the Framework of Expert Recommendations. J Clin Pathol (2021) 74:596–603. doi: 10.1136/jclinpath-2020-206840 32895300

[17] Toss A Piombino C Tenedini E Bologna A Gasparini E Tarantino V. The Prognostic and Predictive Role of Somatic BRCA Mutations in Ovarian Cancer: Results From a Multicenter Cohort Study. Diagnostics (Basel) (2021) 11:565. doi: 10.3390/diagnostics11030565 33801055 PMC8003908

[18] Foundation Medicine: FoundationOne CDx. Available at: https://www.foundationmedicine.com/genomic-testing/foundation-one-cdx (Accessed 2nd December 2021).

[19] Azzollini J Scuvera G Bruno E Pasanisi P Zaffaroni D Calvello M. Mutation Detection Rates Associated With Specific Selection Criteria for BRCA1/2 Testing in 1854 High-Risk Families: A Monocentric Italian Study. Eur J Intern Med (2016) 32:65–71. doi: 10.1016/j.ejim.2016.03.010 27062684

[20] Mandelker D Donoghue M Talukdar S Bandlamudi C Srinivasan P Vivek M. Germline-Focussed Analysis of Tumour-Only Sequencing: Recommendations From the ESMO Precision Medicine Working Group. Ann Oncol (2019) 30:1221–31. doi: 10.1093/annonc/mdz136 PMC6683854 31050713

[21] Banerjee S Moore KN Colombo N Scambia G Kim BG Oaknin A. Maintenance Olaparib for Patients With Newly Diagnosed Advanced Ovarian Cancer and a BRCA Mutation (SOLO1/GOG 3004): 5-Year Follow-Up of a Randomised, Double-Blind, Placebo-Controlled, Phase 3 Trial. Lancet Oncol (2021) 22:1721–31. doi: 10.1016/S1470-2045(21)00531-3 34715071

[22] Kemp Z Turnbull A Yost S Seal S Mahamdallie S Poyastro-Pearson E. Evaluation of Cancer-Based Criteria for Use in Mainstream BRCA1 and BRCA2 Genetic Testing in Patients With Breast Cancer. JAMA Netw Open (2019) 2:e194428. doi: 10.1001/jamanetworkopen.2019.4428 31125106 PMC6632150

[23] Hamilton JG Symecko H Spielman K Breen K Mueller R Catchings A. Uptake and Acceptability of a Mainstreaming Model of Hereditary Cancer Multigene Panel Testing Among Patients With Ovarian, Pancreatic, and Prostate Cancer. Genet Med (2021) 23:2105–13. doi: 10.1038/s41436-021-01262-2 PMC8556289 34257420

[24] Hennessy BT Timms KM Carey MS Gutin A Meyer LA Flake DD 2nd. Somatic Mutations in BRCA1 and BRCA2 Could Expand the Number of Patients That Benefit From Poly (ADP Ribose) Polymerase Inhibitors in Ovarian Cancer. J Clin Oncol (2010) 28:3570–6. doi: 10.1200/JCO.2009.27.2997 PMC2917312 20606085

[25] Pennington KP Walsh T Harrell MI Lee MK Pennil CC Rendi MH. Germline and Somatic Mutations in Homologous Recombination Genes Predict Platinum Response and Survival in Ovarian, Fallopian Tube, and Peritoneal Carcinomas. Clin Cancer Res (2014) 20:764–75. doi: 10.1158/1078-0432.CCR-13-2287 PMC3944197 24240112

[26] Yost S Ruark E Alexandrov LB Rahman N. Insights Into BRCA Cancer Predisposition From Integrated Germline and Somatic Analyses in 7632 Cancers. JNCI Cancer Spectr (2019) 3:pkz028. doi: 10.1093/jncics/pkz028 31360904 PMC6649772

[27] Vos JR Fakkert IE de Hullu JA van Altena AM Sie AS Ouchene H. Universal Tumor DNA BRCA1/2 Testing of Ovarian Cancer: Prescreening PARPi Treatment and Genetic Predisposition. J Natl Cancer Inst (2020) 112:161–9. doi: 10.1093/jnci/djz080 PMC7019087 31076742

[28] Spizzo G Puccini A Xiu J Goldberg RM Grothey A Shields AF. Molecular Profile of BRCA-Mutated Biliary Tract Cancers. ESMO Open (2020) 5(5):e000682. doi: 10.1136/esmoopen-2020-000682 32576609 PMC7312328

[29] Jalloul N Gomy I Stokes S Gusev A Johnson BE Lindeman NI. Germline Testing Data Validate Inferences of Mutational Status for Variants Detected From Tumor-Only Sequencing. JCO Precis Oncol (2021) 5:PO.21.00279. doi: 10.1200/PO.21.00279 34820595 PMC8608266

[30] Liu YL Selenica P Zhou Q Iasonos A Callahan M Feit NZ. BRCA Mutations, Homologous DNA Repair Deficiency, Tumor Mutational Burden, and Response to Immune Checkpoint Inhibition in Recurrent Ovarian Cancer. JCO Precis Oncol (2020) 4:PO.20.00069. doi: 10.1200/PO.20.00069 32923884 PMC7446408

[31] Harpaz N Gatt YE Granit RZ Fruchtman H Hubert A Grinshpun A. Mucinous Histology, BRCA1/2 Mutations, and Elevated Tumor Mutational Burden in Colorectal Cancer. J Oncol (2020) 2020:6421205. doi: 10.1155/2020/6421205 32377194 PMC7196997

[32] Yeo ZX Wong JC Rozen SG Lee AS. Evaluation and Optimisation of Indel Detection Workflows for Ion Torrent Sequencing of the BRCA1 and BRCA2 Genes. BMC Genomics (2014) 15:516. doi: 10.1186/1471-2164-15-516 24962530 PMC4079958

[33] Ruiz A Llort G Yagüe C Baena N Viñas M Torra M. Genetic Testing in Hereditary Breast and Ovarian Cancer Using Massive Parallel Sequencing. BioMed Res Int (2014) 2014:542541. doi: 10.1155/2014/542541 25136594 PMC4098986

[34] Poulet A Privat M Ponelle F Viala S Decousus S Perin A. Improved Efficiency and Reliability of NGS Amplicon Sequencing Data Analysis for Genetic Diagnostic Procedures Using AGSA Software. BioMed Res Int (2016) 2016:5623089. doi: 10.1155/2016/5623089 27656653 PMC5021467

[35] Tao H Liu S Huang D Han X Wu X Shao YW. Acquired Multiple Secondary BRCA2 Mutations Upon PARPi Resistance in a Metastatic Pancreatic Cancer Patient Harboring a BRCA2 Germline Mutation. Am J Transl Res (2020) 12:612–7. PMC7061843 32194909

[36] Tobalina L Armenia J Irving E O’Connor MJ. Forment J.V. A Meta-Analysis of Reversion Mutations in BRCA Genes Identifies Signatures of DNA End-Joining Repair Mechanisms Driving Therapy Resistance. Ann Oncol (2021) 32:103–12. doi: 10.1016/j.annonc.2020.10.470 33091561

[37] Cunningham JM Cicek MS Larson NB Davila J Wang C Larson MC. Clinical Characteristics of Ovarian Cancer Classified by BRCA1, BRCA2, and RAD51C Status. Sci Rep (2014) 4:4026. doi: 10.1038/srep04026 24504028 PMC4168524

[38] Hansmann T Pliushch G Leubner M Kroll P Endt D Gehrig A. Constitutive Promoter Methylation of BRCA1 and RAD51C in Patients With Familial Ovarian Cancer and Early-Onset Sporadic Breast Cancer. Hum Mol Genet (2012) 21:4669–79. doi: 10.1093/hmg/dds308 PMC3471399 22843497

[39] Azzollini J Pesenti C Pizzamiglio S Fontana L Guarino C Peissel B. Constitutive BRCA1 Promoter Hypermethylation Can Be a Predisposing Event in Isolated Early-Onset Breast Cancer. Cancers (Basel) (2019) 11:58. doi: 10.3390/cancers11010058 PMC6356733 30634417

[40] Hu C Hart SN Gnanaolivu R Huang H Lee KY Na J. A Population-Based Study of Genes Previously Implicated in Breast Cancer. N Engl J Med (2021) 384:440–51. doi: 10.1056/NEJMoa2005936 PMC8127622 33471974

[41] Breast Cancer Association Consortium Dorling L Carvalho S Allen J González-Neira A Luccarini C. Breast Cancer Risk Genes - Association Analysis in More Than 113,000 Women. N Engl J Med (2021) 384:428–39. doi: 10.1056/NEJMoa1913948 PMC7611105 33471991

[42] Coignard J Lush M Beesley J O’Mara TA Dennis J Tyrer JP. A Case-Only Study to Identify Genetic Modifiers of Breast Cancer Risk for BRCA1/BRCA2 Mutation Carriers. Nat Commun (2021) 12:1078. doi: 10.1038/s41467-020-20496-3 33597508 PMC7890067

