# Supplementary figures and images for: Management of BRCA Tumour Testing in an Integrated Molecular Tumour Board Multidisciplinary Model
Source: Front Oncol. 2022 Apr 8;12:857515. doi: 10.3389/fonc.2022.857515 (PMC9026437; doi:10.3389/fonc.2022.857515)

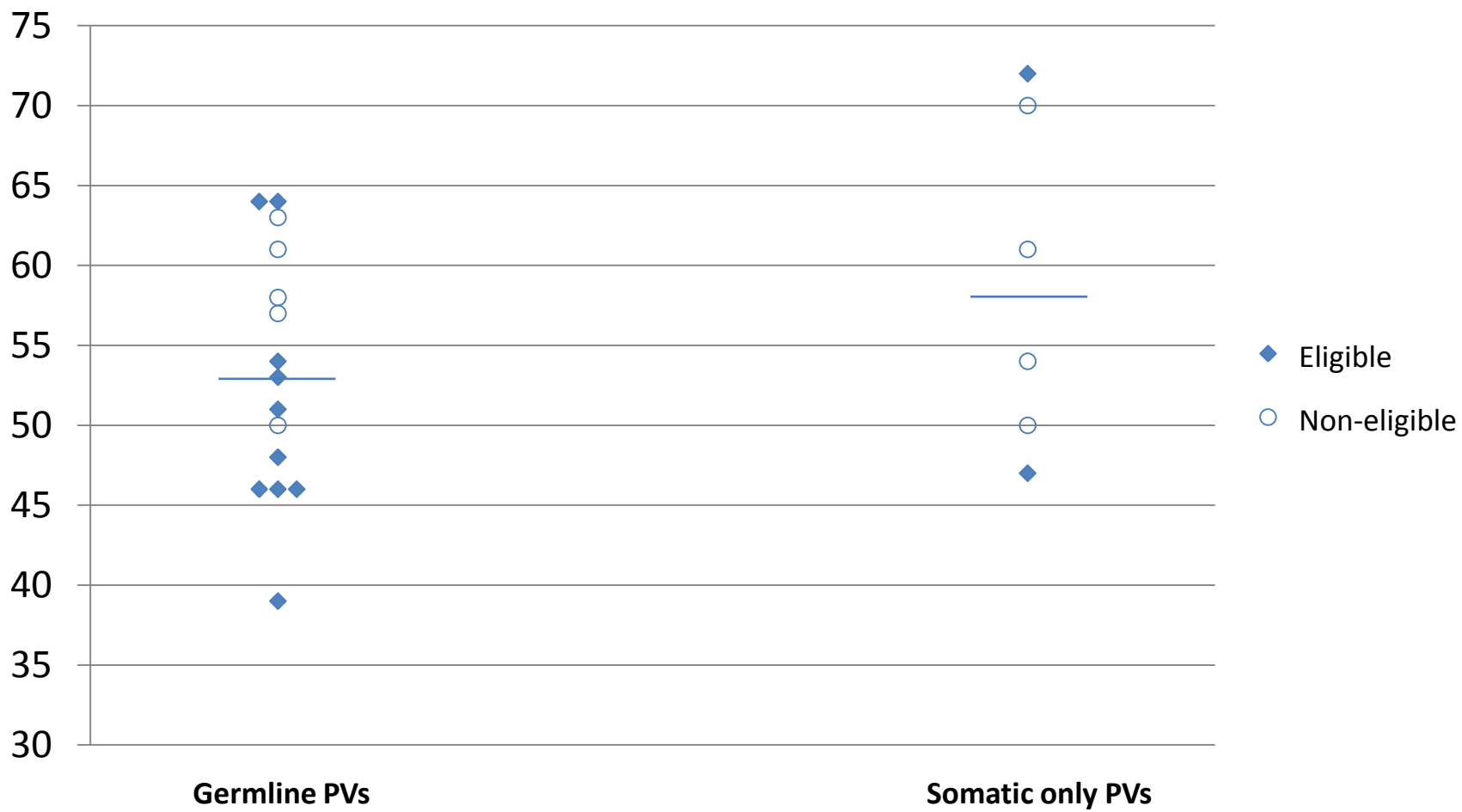

Supplement: Supplementary Figure 1 — Age at diagnosis of ovarian cancer in patients with confirmed germline vs. somatic only BRCA pathogenic/likely pathogenic variants (PVs). The median age is indicated by the horizontal lines; eligibility to germline testing according to the empirical criteria used at our Institution is indicated for each patient. [file Image_1.pdf]
